# Severe Central Nervous System and Respiratory Depression in a Neonate Following Accidental Oral Ingestion of Brimonidine Tartrate

**DOI:** 10.7759/cureus.63124

**Published:** 2024-06-25

**Authors:** Faiza Gul, Mudassir Shah, Muhammad Waqar, Radhika Bassi, Safdar Shah, Nida Sethi

**Affiliations:** 1 Paediatrics, Lady Reading Hospital, Peshawar, PAK; 2 Paediatrics, Hayatabad Medical Complex, Peshawar, PAK; 3 Internal Medicine, Ross University School of Medicine, Bridgetown, BRB

**Keywords:** adrenergic agonist, accidental ingestion, respiratory depression, ophthalmic solution, brimonidine tartrate

## Abstract

Brimonidine is a third-generation alpha-2 adrenergic agonist and is classified as an ocular hypotensive agent. It is used for chronic glaucoma treatment by lowering intraocular pressure, crucial for preventing blindness. Brimonidine works by reducing aqueous humor production and increasing uveoscleral outflow. The improper use of brimonidine in children can result in severe adverse effects. If brimonidine eye drops are ingested orally, it can cause significant depression of the cardiorespiratory and central nervous systems. This is a case report of a 27-day-old neonate, who presented with central nervous system and respiratory depression after accidental ingestion of one drop of brimonidine tartrate ophthalmic solution. On arrival, he was having shallow breathing, a low Glasgow Coma Scale score, pinpoint pupils, and absent deep tendon reflexes. Gastric lavage was performed and supportive treatment was started. The patient showed gradual improvement and completely recovered within 48 hours.

## Introduction

Brimonidine, introduced in 1996, is a selective alpha-2 adrenergic agonist that reduces aqueous humor production and increases uveoscleral outflow [1]. The reduction in aqueous humor is due to vasoconstriction of uveal blood vessels, while the increased outflow results from prostaglandin synthesis induced by alpha-agonist activity, leading to ciliary muscle relaxation [2]. Its lipophilic nature allows for quick absorption through the cornea and passage across the blood-brain barrier, which can impact the central nervous system (CNS) [3]. Notably, CNS depression and apnea have been reported in neonates, making brimonidine unsuitable for children under two years old [4]. Once absorbed, brimonidine is rapidly metabolized, with peak plasma levels occurring one to four hours after administration and a half-life of around two hours [5]. Brimonidine is primarily metabolized in the liver and excreted in the urine [6]. Brimonidine can cause sedation due to its effects on postsynaptic receptors in the locus coeruleus, as well as hypotension and bradycardia, resulting from both central and peripheral actions. Central alpha-2 receptor stimulation may lead to coma, respiratory depression, and miosis [7]. Brimonidine is also used in the treatment of other conditions, such as rosacea, due to its vasoconstrictive properties [8].

In this case report, a 27-day-old neonate was brought in by his parents with a history of one hour of irritability, followed by drowsiness and irregular breathing. The history revealed that the mother had mistakenly given her child anti-glaucoma eye drops (brimonidine tartrate), prescribed for his father, confusing them with colic drops, orally one hour prior to this event. Gastric lavage was performed to prevent further absorption of the drug from the gastrointestinal tract. Other supportive measures were initiated, including oxygen inhalation to improve oxygen saturation, intravenous naloxone administration to counteract CNS depression, and fluid boluses to maintain adequate hydration. All these measures resulted in the patient's improvement. The respiratory rate was increased, neurological status improved as evidenced by a higher Glasgow Coma Scale (GCS) score, and vital signs stabilized, indicating overall recovery.

## Case presentation

**Figure 1:**
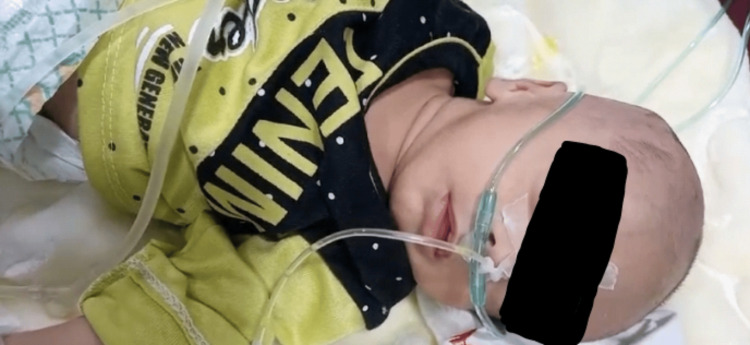
The neonate exhibiting signs of respiratory and central nervous system depression following the administration error.

A previously healthy 27-day-old neonate was brought to the pediatric emergency department with drowsiness and shallow breathing (Figure 1). The neonate was born via cesarean section at 35 weeks of gestation, with a birth weight of 2.9 kg. His past medical history was unremarkable except for colic, for which colic drops were prescribed by a doctor two days prior. His mother mistakenly gave him one drop of the anti-glaucoma drug brimonidine tartrate (0.2%) an hour before the event, prescribed for his father’s glaucoma, confusing it with the colic drops (Appendix).

The neonate arrived by ambulance, presenting with shallow breathing, lethargy, and drowsiness. On general physical examination, he exhibited CNS and respiratory depression. He was lethargic, unarousable, hypotonic, and pale. His weight was 4 kg, and his vital signs included a respiratory rate of 17 breaths per minute (reference range: 30-60 in neonates), SpO2 (peripheral oxygen saturation) of 84% (reference range: 91-95%), temperature of 96°F (reference range: 96.8-99.5), blood pressure of 60/50 mmHg (reference range: systolic 60-80, diastolic 30-50), and a heart rate of 140/minute (reference range: 100-165). Chest examination was normal with no added breath sounds. The cardiovascular examination was normal, with a pulse rate of 140/minute (reference range: 100-160 beats per minute), normal heart sounds, palpable peripheral pulses, and adequate capillary refill time, indicating stable cardiovascular function. Eye examination showed bilateral miosis and absent pupillary light reflex. Neurological examination revealed a low GCS score of 8/15, lethargy, decreased deep tendon reflexes, and decreased response to pain.

Laboratory results were normal except for persistent hyperglycemia and 2+ glucose in urine. Complete blood count, electrolytes, liver function tests, renal function tests, arterial blood gases, C-reactive protein (CRP), blood ketones, prothrombin time (PT), and activated partial thromboplastin time (aPTT) were normal (Table 1). Ultrasound of the skull and chest X-ray were normal. Urine toxicology was negative.

**Table 1:** Laboratory results of the neonate. PCO2: partial pressure of carbon dioxide; HCO3: bicarbonate.

Parameter	Value	Units	Reference range
White blood cells	6.7	x 10^9/L	4-12
Hemoglobin	13.5	g/dL	11.5-17.5
Platelet	270	x 10^9/L	150-450
Calcium	9	mmol/L	8-10
Sodium	136	mmol/L	135-150
Potassium	4.2	mmol/L	3.5-5.1
Chloride	104	mmol/L	96-112
Random blood glucose	10.6	mmol/L	3.9-5.5
C-reactive protein	0.1	mg/dL	0.1
pH	7.35		7.35-7.45
PCO2	40	mmHg	35-45
HCO3	24	mmol/L	22-26

The neonate was admitted to the pediatric unit for three days and closely observed for respiratory depression. He received supportive care, including oxygen therapy, intravenous fluids, and monitoring of vital signs. Continuous positive airway pressure (CPAP) was used with an initial pressure setting of 5 cm H2O, which was gradually reduced as his condition improved. The oxygen flow rate was also gradually reduced from 6 liters per minute to 1 liter per minute. After three days, he was discharged home with normal vital signs, including a respiratory rate of 40 breaths per minute.

## Discussion

Brimonidine, an imidazole derivative, is an alpha-2 selective adrenergic agonist similar to clonidine, which is known for its toxicity in children [3]. Clonidine acts on alpha-2 adrenergic and imidazole receptors to lower blood pressure, making it useful for hypertension and tachycardia control. Brimonidine's structure is similar to clonidine but is significantly more selective for alpha-2 receptors, being up to 12 times more selective than clonidine and up to 32 times more selective than apraclonidine [6]. This results in comparable effects but with less systemic toxicity due to lower lipid solubility. Brimonidine is used to treat open-angle glaucoma and ocular hypertension by reducing aqueous humor production and increasing its elimination through uveoscleral outflow [9]. The reduction is due to vasoconstriction of uveal blood vessels, while the increased outflow results from prostaglandin synthesis induced by alpha-agonist activity, leading to ciliary muscle relaxation [2]. Brimonidine is primarily metabolized in the liver [10], with peak plasma levels occurring one to four hours after administration. The half-life of brimonidine is around two hours and its metabolites are excreted by the kidneys [11]. In children, the incomplete development of the blood-brain barrier and the drug’s high lipophilicity can result in significant CNS depression, leading to symptoms such as fatigue, weakness, lethargy, apnea, bradycardia, hypotension, respiratory depression, and coma [12]. High doses of brimonidine, typically exceeding the therapeutic range used for ocular applications, can induce oxidative stress in neuronal cells by disrupting the balance of reactive oxygen species, damaging proteins, lipids, and DNA [13]. Although the exact amount defining a high dose varies, ingestion of amounts higher than the recommended ocular dose (0.1-0.2% solution, one drop in each eye, typically totaling around 0.05 mg) is considered hazardous, particularly in neonates and young children due to their increased susceptibility to CNS effects. Brimonidine may also modulate glutamate neurotransmission, potentially leading to neuronal damage [14]. Additionally, it can affect calcium homeostasis, contributing to neuronal dysfunction and death, and may influence inflammatory responses in the CNS, which are linked to various neurological disorders [15].

Gastric lavage is a procedure that has been used historically in cases of toxic ingestion, especially when the ingestion is recent (within one hour). Gastric lavage involves inserting a tube into the stomach and repeatedly flushing it with saline or water to remove ingested substances. In neonates and infants, the use of gastric lavage is controversial due to the risks associated with the procedure, such as aspiration and perforation. For brimonidine tartrate, there is limited literature specifically addressing its use in neonates. However, similar management strategies have been noted in pediatric toxicology references, suggesting that gastric lavage can be employed in severe cases of ingestion of CNS depressants when performed with caution and appropriate expertise [16]. Naloxone is an opioid antagonist typically used to reverse opioid overdose. However, it has been reported to have some benefits in cases of clonidine and other alpha-2 adrenergic agonist toxicity, including brimonidine, due to its ability to counteract CNS depression [17]. While the exact mechanism by which naloxone exerts its effects in non-opioid toxicities is not fully understood, its use in this case was justified given the severity of the symptoms and the need for rapid intervention. The decision to administer naloxone was based on its potential to improve respiratory function and level of consciousness in the context of CNS depression caused by alpha-2 adrenergic agonist toxicity. Brimonidine is also used in the treatment of other conditions, such as rosacea. In 2013, the FDA approved a topical formulation of brimonidine gel (Mirvaso) for the treatment of persistent facial erythema of rosacea in adults. This demonstrates the versatility of brimonidine in managing different conditions due to its vasoconstrictive properties [8].

In this case, the neonate presented with symptoms of CNS and respiratory depression. Differential diagnoses for altered consciousness in neonates include hypoglycemia, intracranial hemorrhage, infection (such as meningitis or sepsis), metabolic disorders, and drug toxicity. The absence of infection markers and normal imaging studies supported the diagnosis of drug-induced CNS depression. The medication error that occurred due to similar packaging of both brimonidine and colic drops can be prevented in the future through proper medication storage and labeling, as well as through the education of caregivers.

## Conclusions

This case report highlights the significant risk of severe CNS and respiratory depression in neonates following accidental oral ingestion of brimonidine tartrate ophthalmic solution. The rapid onset of symptoms, including lethargy, shallow breathing, and low GCS, underscores the drug's potent effects on the immature neonatal system. Prompt interventions, such as gastric lavage, oxygen therapy, naloxone administration, and CPAP, were crucial in the successful management and recovery of the neonate. This case emphasizes the critical need for careful medication handling and awareness of potential toxicities in the pediatric population.

## Figures and Tables

**Figure 2:**
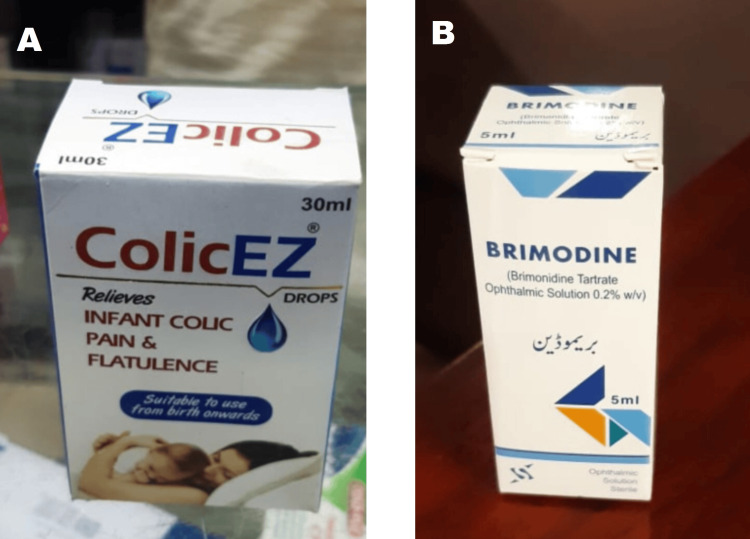
Comparison of the outer packaging of anti-glaucoma drops and colic drops, highlighting the similarities that led to the administration error. A: Outer packaging of the colic drops. B: Outer packaging of the brimonidine drops (anti-glaucoma).
